# Morphological, anatomical, and bioactive properties of *Hypericum scabrum* L.: effects on diabetes mellitus, Alzheimer’s disease, and HDFa fibroblasts and U87-MG cancer cells

**DOI:** 10.1007/s00709-025-02037-1

**Published:** 2025-01-30

**Authors:** Sena Öner, Abdulrahim Kadı, Enes Tekman, Ayşe Cemre Kararenk, Elif Beyza Özer, Kübra Nalkıran Ergin, Hafize Yuca, Mehmet Enes Arslan, Resul Duman, Aydan Acar Şahin, Nur Münevver Pinar, Alptuğ Atila, Gülnur Ekşi Bona, Songül Karakaya

**Affiliations:** 1 Department of Molecular Biology and Genetics, Faculty of Science, Erzurum Technical University, Erzurum, Türkiye; 2 Department of Pharmaceutical Botany, Faculty of Pharmacy, Atatürk University, Erzurum, Türkiye; 3 Ankara University Graduate School of Health Sciences, Ankara, Türkiye; 4 Department of Pharmacognosy, Faculty of Pharmacy, Atatürk University, Erzurum, Türkiye; 5 Department of Biology, Faculty of Science, Ankara University, Ankara, Turkey; 6 Department of Analytical Chemistry, Faculty of Pharmacy, Atatürk University, Erzurum, Türkiye; 7 Department of Pharmaceutical Botany, Faculty of Pharmacy, İstanbul-Cerrahpaşa University, İstanbul, Türkiye

**Keywords:** Diabetes mellitus, HDFa, U87-MG, *Hypericum scabrum*, Cancer, Alzheimer’s disease, Element, Phenolic, Amino acid

## Abstract

Diabetes mellitus (DM) and cancer are multifactorial diseases with significant health consequences, and their relationship with aging makes them particularly challenging. Epidemiological data suggests that individuals with DM are more susceptible to certain cancers. This study examined the bioactive properties of *Hypericum scabrum* extracts, including methanol, hexane, and others, focusing on their inhibitory effects on key enzymes associated with DM and neurodegenerative diseases, such as acetylcholinesterase, butyrylcholinesterase, *α*-amylase, and *α*-glucosidase. Additionally, the impact of these extracts on human fibroblast (HDFa) and glioblastoma (U87MG) cancer cells was evaluated. The methanol extract was analyzed for elemental composition using ICP-MS, secondary metabolites, and amino acids via LC–MS/MS and underwent morphological and anatomical characterization. The methanol extract demonstrated notable inhibitory activity, with an IC_50_ value of < 1 µg/mL against *α*-glucosidase, surpassing acarbose in efficacy. The flower essential oil exhibited the highest inhibition (79.95%) of butyrylcholinesterase and the strongest acetylcholinesterase inhibition (21.62%). Elemental analysis revealed high concentrations of Na and K, while quinic acid and proline were identified as major metabolites, with proline concentrations reaching 494.0482 nmol/mL in the aerial part extract. The anticancer assays revealed higher cytotoxicity in U87MG glioblastoma cells compared to HDFa fibroblasts, suggesting potential applications for cancer therapy. The plant grows 20–50 cm tall, with yellow flowers and ovoid-ribbed capsules containing brown, reniform seeds. Its leaves are amphistomatic and ornamented, while stems feature striate cuticles and paracytic stomata. The pollen grains are microreticulate with syncolporate apertures. These results underscore the promising therapeutic potential of *H. scabrum* in managing DM, cancer, and neurodegenerative diseases, with its ability to inhibit key enzymes and show selective cytotoxicity against cancer cells.

**Graphical abstract:**

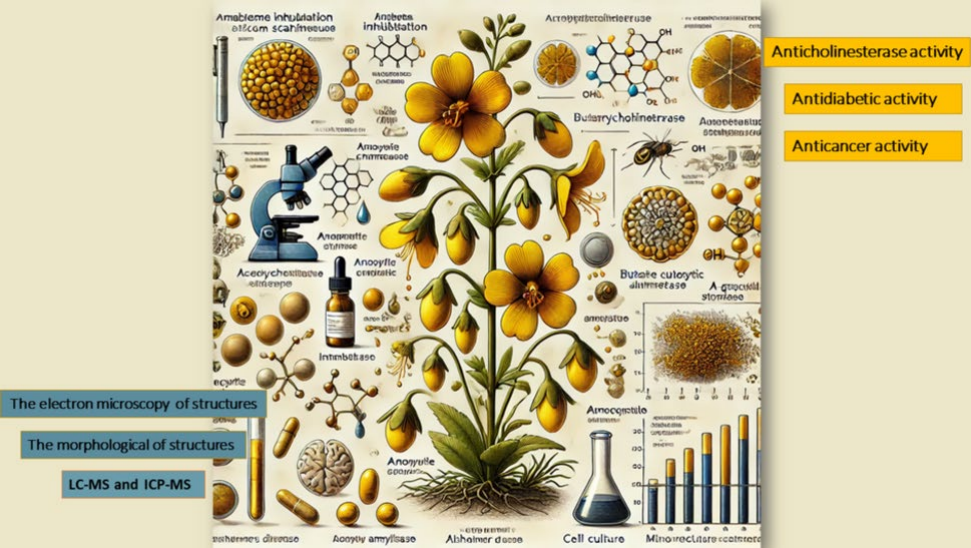

## Introduction

Currently, there are 55.2 million individuals grappling with dementia globally, and projections indicate a surge to 139 million by 2050 as the global population ages. Dementia stands out as a paramount public health concern and one of the foremost challenges in healthcare. Alzheimer’s disease (AD) takes the lead as the predominant cause of age-related dementia, encompassing 60–70% of reported cases. Exploring risk reduction strategies in susceptible populations could serve as a valuable supplement to conventional drug discovery approaches, offering a proactive means to prevent AD incidence and alleviate the strain on both families and society (Luo et al. 2023).

Diabetes mellitus (DM) is a multifaceted metabolic condition marked by elevated blood glucose levels and linked to complications affecting small and large blood vessels, such as retinopathy, nephropathy, neuropathy, and cardiovascular issues (Sims-Robinson et al. 2010).

The term “cancer” encompasses a spectrum of over a hundred conditions impacting various anatomical regions. Cancer represents a multifaceted progression involving the uncontrollable and rapid proliferation of bodily cells. The development of cancer is gradual and evolves into an invasive state through sequential stages, complicating the early identification and prevention efforts. Globally, it poses a substantial health challenge attributed to the absence of effective early detection techniques (Tariq et al. 2017).

DM has been associated with various adverse health effects, including cognitive impairment. The connection between impaired cognitive functions and DM suggests a potential contribution to AD by DM (Arvanitakis et al. 2004). DM is physiologically linked to AD; thus, insulin resistance and hyperinsulinemia associated with the central nervous system can lead to cognitive impairment. According to a meta-analysis of consolidated scientific findings, individuals with type 2 diabetes have a 56% increased risk of displaying Alzheimer’s symptoms (Amir Rawa et al. 2022).

The prevalence of both DM and AD has been steadily increasing, creating significant challenges for societies and health organizations. Although these conditions were once considered separate, emerging evidence suggests they are interconnected. Shared factors like chronic inflammation, oxidative stress, mitochondrial dysfunction, adiponectin deficiency, altered cholinesterase activity, and vascular damage may explain the common coexistence of DM and AD in many patients (Janoutová et al. 2022). DM and cancer are complex, severe conditions with multiple causes. Their high prevalence means even minor interactions between the two can have major effects. Epidemiological studies consistently show an increased cancer risk in individuals with diabetes, along with a moderate rise in mortality rates (Vigneri et al. 2009). AD and diabetes are closely linked, with 25–35% of AD patients also having type 2 diabetes (T2D). Diabetes increases the risk of developing AD by 50–60%, especially in those with earlier onset or longer duration. Despite this connection, effective treatments for AD remain lacking and the high comorbidity complicates its prevention and treatment (Yuan et al. 2023).

Cancer and AD share an association with the aging process, yet they seldom manifest simultaneously (Nixon 2017). AD is intricately linked to type 2 diabetes mellitus (T2D), with both conditions exhibiting overlapping pathophysiological mechanisms. This correlation has spurred the hypothesis that AD could be deemed as a form of T2D (Ajiboye et al. 2018).

Throughout the annals of history, botanical remedies have woven an indispensable tapestry in humanity’s quest for healing, shaping the very landscape of modern medicine. Remarkably, approximately 60% of the arsenal deployed against cancer traces its roots back to nature’s repository, highlighting the transformative influence of plant-derived compounds on contemporary therapeutic modalities (Solowey et al. 2014;). The prevalence of DM is on a steep incline, with projections indicating a further surge by 2030. Beyond conventional therapeutic avenues, a plethora of herbal remedies are advocated for its management. Herbal medicines have garnered longstanding acclaim in DM treatment due to their propensity for minimal or absent side effects (Nasri et al. 2015).

Plants exhibiting favorable impacts on cognitive impairments, coupled with robust acetylcholinesterase inhibitory, anti-inflammatory, and antioxidant properties, hold promise for clinical consideration in AD therapy (Penido et al. 2017).

While certain lexicographers have endeavored to trace its origins to hypo- or hyper-ericum (below or above the heath), the etymology of *Hypericum* remains unequivocal. Ancient Greeks bestowed this name upon a plant or plants suspended above their sacred icons to repel malevolent forces, elucidating its significance and derivation (Ernst 2003). Hypericin and hyperforin are compounds in *H. perforatum* and *H. scabrum* and are naphthodianthrone. Hypericin is highly photoreactive, absorbing light and inducing photosensitization and cytotoxicity. When exposed to oxygen and light, hypericin generates reactive oxygen species (ROS), leading to cell and animal toxicity. Notably, in vitro studies also demonstrate its antiproliferative effects, even without light exposure. Hypericin’s photodynamic antimicrobial effects on pathogenic fungi and its potential in photodynamic therapy for human cancer cells are well recognized. The antitumor effects of *H. perforatum* and its active compound hyperforin have been reported. They inhibit tumor growth by targeting inflammatory mediators, pro-survival kinases, and angiogenesis. Hyperforin disrupts mitochondrial function, reduces ROS production, and prevents tumor cell proliferation. It also alters cell pH, reducing metastasis and inducing apoptosis in cancer cells, showing strong potential for cancer prevention and treatment (Sytar et al. 2021, 2016; Ayan et al. 2009; Saberi et al. 2024; Menegazzi et al. 2020).

The historical usage of *Hypericum* species in medicine traces back over 2400 years. Among the populace of Anatolia, Turkey, *H. scabrum* L., belonging to the Hypericaceae family, is known by various names including “mayasıl otu,” “kepir otu,” and “kızılcık otu.” *H. scabrum* is prevalent not only in Turkey but also in several countries including Afghanistan, Iran, Iraq, Syria, Azerbaijan, and Pakistan. Within Turkey, *H. scabrum* thrives in various cities such as Hakkari, Siirt, Kastamonu, Elazig, Erzurum, Sivas, Van, Bayburt, Antalya, and Ankara (Ergin et al. 2022). It is a perennial herb native to Turkey and grows in dry, rocky slopes, and open woodlands. It has long been used in Turkish folk medicine for its antispasmodic, sedative, and anti-inflammatory properties. A topical ointment made from its aerial parts is traditionally used for treating psoriasis. Like other *Hypericum* species, it has a prominent role in traditional medicine, treating various conditions such as hepatitis, cystitis, chronic gastritis, ulcers, wounds, hemorrhoids, and constipation. Scientific studies highlight its antispasmodic, anti-inflammatory, sedative, and antidepressant effects (Keser et al. 2020; Ayan et al. 2009). Natural products from the *Hypericum* genus, such as *H. humifusum* and *H. perfoliatum*, exhibit a range of pharmacological properties. Specifically, *H. humifusum* demonstrated significant enzyme inhibitory effects, including acetylcholinesterase (3.86–4.57 mg GALAEs/g), *α*-glucosidase (0.73–2.55 mmol ACEs/g), and *α*-amylase (3–8 mmol ACEs/g) activities, highlighting its potential for therapeutic applications (Béjaoui et al. 2017).

The study comprehensively examined the inhibitory effects of various extracts and sub-extracts from different parts of *Hypericum scabrum*, including methanol, hexane, dichloromethane, ethyl acetate, butanol, and aqueous extracts. It evaluated their impact on the enzymes acetylcholinesterase and butyrylcholinesterase, as well as their inhibitory activities against *α*-amylase and *α*-glucosidase. The research also investigated the effects of *H. scabrum* on HDFa fibroblast and U87-MG cancer cells. Additionally, the methanol extract was analyzed for 21 elements using ICP-MS, and quantitative assessments of certain secondary metabolites and 43 amino acids were conducted via LC–MS/MS. Morphological and anatomical studies of the plant were also included in the analysis.

## Material and method

### Plant material

*H. scabrum* was initially identified in 2016 by Prof. Dr. Hayri Duman (Ergin et al. 2022). Subsequent to its discovery, study samples were procured from Palandöken Mountain, adjacent to Abdurrahman Gazi Tomb, and Erzurum Urban Forest during the months of June through August in both 2019 and 2020. The specimens were gathered from mountain slopes at altitudes ranging from 1910 to 1990 m during the plant’s flowering and fruiting phases. Herbarium specimens, labeled with the AUEF 1278 identifier, are meticulously preserved at the Biodiversity Application and Research Center of Atatürk University.

### Extraction and fractionation

The extraction and sub-extraction procedures employed in this study were conducted following the detailed methodology outlined in Ergin et al. (2022). Individual parts of *H. scabrum* were carefully powdered and subjected to maceration in methanol at room temperature. This process was carried out using a mechanical mixer for 8 h daily over a period of 3 days to ensure maximum extraction efficiency. After maceration, the methanolic extracts were filtered, and the solvent was evaporated under reduced pressure to obtain a dry extract. The dry residue was subsequently reconstituted in a methanol-to-water mixture (1:9, v/v) to enhance solubility and facilitate fractionation. Sequential liquid–liquid partitioning was then performed using solvents of increasing polarity, including *n*-hexane, dichloromethane, ethyl acetate, and *n*-butanol, to yield distinct sub-extracts. Each sub-extract was carefully separated and concentrated using rotary evaporation to remove the solvents, leaving behind a residue representing each fraction. The weights of these sub-extracts were recorded for further analysis. This systematic approach ensured a comprehensive extraction of the bioactive constituents from *H. scabrum*.

#### Elemental analysis

Elemental concentrations in the solution were meticulously analyzed using an inductively coupled plasma-mass spectrometer (ICP-MS), specifically the Agilent 7800 series, manufactured by Agilent Technologies, Japan. For sample introduction, a glass MicroMist nebulizer from the U-series in Australia was employed, in conjunction with a double-pass quartz spray chamber from the USA, ensuring efficient aerosol formation and sample delivery into the plasma. The plasma system consisted of a quartz torch (2.5 mm diameter) and nickel components, including sample and skimmer cones, optimized for the x-lens to improve ion transmission and sensitivity. To ensure accuracy and prevent contamination, all quartz and nickel parts were subjected to stringent cleaning procedures. The quartz and glass components were soaked in a 5–10% nitric acid (HNO_3_) solution overnight, followed by thorough rinsing with distilled water, drying, and careful reinstallation. Nickel components were subjected to ultrasonic cleaning in pure water, a 5% HNO_3_ solution, and distilled water, each for 5 min. After cleaning, the nickel parts were rinsed thoroughly, dried, and reassembled. Prior to analysis, a 45-min helium gas purge was performed on the system to eliminate any residual gases and ensure a stable environment. The instrument was then calibrated using a tuning solution, and the calibration curves were verified within the standard reference range for various elements, ensuring the accuracy of measurements. Sequential tests were conducted to confirm the optimal performance of the system, with parameter adjustments made to fine-tune the instrument’s operation. Sample analysis was carried out by loading the prepared samples via an autosampler, followed by a thorough washing of the autosampler, tubing, and probe section to eliminate any cross-contamination between samples. Measurements were conducted under carefully controlled conditions, including RF power, carrier gas flow, and nebulizer pump speed. Argon gas was used as the carrier gas to efficiently transport the aerosolized sample into the plasma, where ionization and subsequent mass spectrometric analysis took place (Tekman et al. 2024).

### Quantitative analysis of secondary metabolites

The quantitative analysis of secondary metabolites in the most potent extracts was conducted using the Agilent 6460 Triple Quadrupole liquid chromatography-tandem mass spectrometer system (LC–MS/MS), located at the Atatürk University East Anatolia High Technology Application and Research Center (DAYTAM). This sophisticated system combines chromatographic and mass spectrometric techniques to provide accurate and precise quantification of compounds. Separation of the analytes was achieved using an Agilent Poroshell 120 EC-C18 column (4.6 × 100 mm, 3.5 µm), coupled with an Agilent 1260 high-performance liquid chromatography (HPLC) system. The system operated in positive ion mode, employing electrospray ionization (ESI) to generate protonated ions [M + H]^+^, which were then detected and quantified. The analysis was carried out in standard scan mode for each compound at its respective concentration, allowing for precise identification and quantification of metabolites. For optimal chromatographic separation, a dual carrier phase was employed, with Phase A consisting of 0.5% formic acid in water and Phase B consisting of 0.5% formic acid in acetonitrile. The system was calibrated using specific gradients to ensure efficient separation of the analytes. An injection volume of 5.0 µL was used for each sample, and the analysis was conducted in multiple reaction monitoring (MRM) mode. This mode enhances sensitivity and specificity by monitoring transitions between specific precursor and product ions, making it ideal for the quantification of secondary metabolites with high precision. The combination of these advanced techniques enabled the thorough and reliable analysis of secondary metabolites, providing valuable insights into their composition and concentration in the most potent extracts (Yuca et al. 2023).

### Amino acid measurement analysis by LC–MS/MS

Samples were aliquoted into Eppendorf tubes, with 50 µL of each sample dispensed for both experimental and control groups. To each tube, 50 µL of internal standard was added to facilitate accurate quantification. The tubes were then vortexed for 10 s to ensure thorough mixing and a homogenous distribution of the internal standard. Subsequently, 700 µL of the amino acid solvent solution, prepared by mixing mobile phases A and B in a volumetric ratio of 1:4, was introduced into each tube. After the addition of the solvent, each sample was vortexed for 1 min to ensure complete dissolution and uniform distribution of the solvent within the sample. Following the vortexing step, the samples were centrifuged at 4 °C for 8 min to separate the cellular debris from the solution. The resulting supernatant was carefully collected and filtered through a 0.45-µm filter to remove any remaining particulate matter. The filtered supernatant was then ready for analysis using the LC–MS/MS system (Agilent 6460 Triple Quadrupole, USA), ensuring that only the clarified sample reached the instrument for precise quantification and identification of metabolites.

### Cell cultures

The HDFa human fibroblast cell line (ATCC® PCS-201–012™) was cultured to full confluence in Dulbecco’s Modified Eagle Medium (DMEM) supplemented with 1% penicillin/streptomycin antibiotics and 10% fetal bovine serum (FBS). The cells were maintained in a 5% CO_2_ incubator at a constant temperature of 37 °C, providing the optimal environment for their growth and proliferation. Similarly, the human glioblastoma cell line (U87MG, ATCC® HTB-14™) was cultured in DMEM medium (Gibco®) enriched with 10% FBS (Sigma-Aldrich®) and 1% penicillin/streptomycin (Sigma-Aldrich®). The cells were maintained in conditions that promoted confluence between 80 and 90%, which was considered ideal for preparing them for subsequent experimental procedures. Once the HDFa and U87MG cells reached the desired confluence, the HDFa cells were harvested by dislodging them from their growth surface using a trypsin/EDTA solution (Sigma-Aldrich®). This enzymatic treatment facilitated the detachment of the cells, which were then collected and transferred to a fresh culture medium. The harvested cells were subsequently seeded into 48-well plates in preparation for the experimental assays. These conditions ensured optimal cell density and health for the following experiments.

### Cytotoxicity analyses

Cell viability was assessed using the MTT assay (3-(4,5-dimethylthiazol-2-yl)−2,5-diphenyltetrazolium bromide), a colorimetric method that quantifies cellular metabolic activity. For this assay, cells were seeded into 48-well plates, with each well containing 100 μL of culture medium and 10^5^ cells, both in the presence and absence of the treatment samples. After seeding, the plates were incubated for 24 h at 37 °C in a CO_2_ incubator to allow the cells to adhere and proliferate. Following the incubation, 10 μL of MTT solution (provided by Cayman Chemical Company®) was added to each well. The plates were gently shaken for 1 min to ensure proper mixing of the MTT solution with the cells, after which they were incubated for an additional 3–4 h at 37 °C. During this incubation, viable cells reduced MTT to dark blue formazan crystals, a direct indicator of cellular metabolic activity. After the incubation period, the culture medium was carefully aspirated from each well, ensuring that the formazan crystals at the base of the wells were undisturbed. In cases involving non-adherent cells, the plates were centrifuged at 400 × *g* for 10 min to facilitate cell sedimentation, after which the supernatant was removed. To solubilize the formed formazan crystals, 100 μL of dimethyl sulfoxide (DMSO) was added to each well. The absorbance of the resulting solution, proportional to the amount of formazan formed, was measured at 570 nm using a microplate spectrophotometer. This absorbance value was used to determine the cell viability by comparing the treated samples to the control group.

### Nuclear abnormality investigations

For the detection of mutated nuclei, Hoechst 33258 fluorescent dye was utilized to assess changes in nuclear morphology following exposure to plant-derived compounds. Fibroblast cells were treated with varying concentrations of the compounds, while a culture group receiving no treatment served as the negative control. After a 24-h incubation period, the cells were fixed in a 4% paraformaldehyde solution prepared in phosphate-buffered saline (PBS) at 4 °C for 30 min to preserve cellular and nuclear integrity. Once fixation was completed, the cells were thoroughly washed with PBS to remove any residual fixative. Subsequently, the cells were stained with 1 µM Hoechst 33258 dye, a fluorescent dye that specifically binds to DNA, for a period of 5 min at room temperature. This allowed for the visualization of nuclear alterations, such as fragmentation, condensation, or other structural abnormalities indicative of mutagenic or genotoxic effects. After staining, the cells were rinsed to remove any unbound dye, and the stained nuclei were examined under a Leica® DM IL LED fluorescence microscope. The microscope’s fluorescent capabilities allowed for the clear visualization and documentation of changes in nuclear morphology, which were indicative of any mutagenic or genotoxic effects induced by the plant-derived compounds. The observations were recorded for further analysis of potential DNA damage.

### α-Glucosidase inhibition assay

The *α*-glucosidase inhibitory activity was evaluated following a modified protocol based on the method outlined by Bachhawat et al. (2011) and further referenced by Yuca et al. (2021). The test samples and the positive control, acarbose, were dissolved in a 50 mM potassium phosphate buffer, adjusted to pH 6.9, ensuring an optimal environment for enzyme activity. In a 96-well microplate, 20 μL of the test sample solution was added to each well, followed by 10 μL of *α*-glucosidase enzyme solution (1 U/mL) and 50 μL of the phosphate buffer. The mixture was incubated at 37 °C for 5 min to allow for proper enzyme–substrate interactions. The enzymatic reaction was then initiated by the addition of 20 μL of 3 mM p-nitrophenyl-α-D-glucopyranoside, a synthetic substrate for *α*-glucosidase. The reaction was allowed to proceed for 30 min at 37 °C, during which the enzyme hydrolyzed the substrate, releasing p-nitrophenol, a yellow-colored product that can be quantitatively measured. To halt the reaction, 50 μL of 0.1 M sodium carbonate solution was added to each well, which also serves to stabilize the released p-nitrophenol. Acarbose, a well-known *α*-glucosidase inhibitor, was used as the standard reference compound to compare the inhibitory effects of the test samples. The absorbance of p-nitrophenol at 405 nm was measured using a microplate reader, with the intensity of the absorbance being directly proportional to the concentration of p-nitrophenol and, therefore, the enzymatic activity. The percentage of *α*-glucosidase inhibition was calculated using the following formula: (1 − Δ*A*_405sample_/Δ*A*_405control_) × 100.

### α-Amylase inhibition assay

The inhibitory activity of test samples on *α*-amylase was assessed using a modified protocol adapted from Nampoothiri et al. (2011), as outlined by Yuca et al. (2021). Both the test compounds and the positive control, acarbose, were dissolved in dimethyl sulfoxide (DMSO) to ensure solubility and accurate assay conditions. A 100 μL aliquot of each test sample or acarbose solution was mixed with 100 μL of a freshly prepared 1% starch solution, prepared in a 20 mM sodium phosphate buffer (pH 6.9) containing 6 mM sodium chloride to maintain enzyme stability and optimal reaction conditions. The reaction mixture was incubated at 25 °C for 10 min to allow initial interaction between the starch and the test compounds. After the initial incubation, 100 μL of *α*-amylase solution (0.5 mg/mL) was added, and the reaction was allowed to proceed for an additional 10 min under the same temperature. To stop the enzymatic reaction, 100 μL of dinitrosalicylic acid (DNS) color reagent was added to each well, and the mixture was heated in a boiling water bath for 5 min to develop a colored complex indicative of reducing sugars released from starch hydrolysis. After cooling to room temperature, the absorbance of the reaction mixture was measured at 540 nm using a microplate reader. Acarbose, a standard *α*-amylase inhibitor, was used as a reference to evaluate the efficacy of the test samples. The percentage inhibition of *α*-amylase activity was calculated using the formula: (1 − Δ*A*_540sample_/Δ*A*_540control_) × 100.

### Acetylcholinesterase (AChE) and butyrylcholinesterase (BChE) inhibition assay

The inhibitory activities against acetylcholinesterase (AChE) and butyrylcholinesterase (BChE) were evaluated using a modified version of the method described by Ingkaninan et al. (2000), with slight adjustments based on Karakaya et al. (2023). Both the test samples and the positive control, donepezil, were dissolved in dimethyl sulfoxide (DMSO) to ensure solubility and uniformity in reaction conditions. The assay was conducted in 96-well microplates. For each well, a reaction mixture was prepared by combining 125 µL of 5,5′-dithiobis-(2-nitrobenzoic acid) (DTNB, commonly known as Ellman’s reagent) with 25 µL of the respective substrate solution—acetylthiocholine iodide for AChE or butyrylthiocholine iodide for BChE. This was followed by the addition of 50 µL of Tris–HCl buffer (50 mM, pH 8.0), which maintained the optimal pH for enzymatic activity. Subsequently, 25 µL of the test sample solution or control (donepezil) was introduced into each well, and the mixture was gently agitated to ensure homogeneity. After a brief pre-incubation period, 25 µL of AChE or BChE enzyme solution was added to the wells to initiate the enzymatic reaction. The plate was incubated at room temperature for a specific duration (typically 15–30 min), allowing sufficient time for substrate hydrolysis. The reaction was monitored by measuring the production of the yellow-colored chromophore, 5-thio-2-nitrobenzoic acid, resulting from the reaction of thiocholine with DTNB. Absorbance was recorded at 405 nm using a microplate spectrophotometer. Donepezil was used as the standard reference inhibitor to compare the efficacy of the test samples. The percentage inhibition of enzyme activity was calculated using the formula: (1 − Δ*A*_405sample_/Δ*A*_405control_) × 100.

### Morphological analysis

Morphological and anatomical analyses were conducted using a Leica S8APO stereo microscope, which provided detailed visualization for accurate identification and examination of plant structures. Ten individual specimens of *H. scabrum* were carefully selected for these studies to ensure comprehensive and representative observations. The morphological examination focused on various plant parts, including stems, leaves, flowers, fruits, and roots. For each component, specific attributes such as shape, size, color, surface texture, and structural features were meticulously recorded. Observations were made under different magnifications to capture both macroscopic and microscopic details. The anatomical study involved preparing thin sections of the plant tissues using a microtome or razor blade, depending on the part being analyzed. These sections were stained with appropriate dyes (e.g., safranin or toluidine blue) to enhance the visibility of cellular and tissue structures. The sections were mounted on glass slides with a mounting medium to facilitate detailed examination under the microscope. Key anatomical features such as vascular bundles, epidermal cells, trichomes, stomatal structures, and parenchymatous tissues were identified and documented. Photographic documentation and illustrative diagrams were created for each plant part, highlighting distinguishing morphological and anatomical characteristics. Measurements and descriptive data were compiled to provide a thorough characterization of the species, contributing valuable information for taxonomic and botanical studies of *H. scabrum*.

### Microscopic analysis

Scanning electron microscopy (SEM) analysis was carried out at the Institute of Mineral Research and Exploration Laboratory to examine the micromorphological characteristics of the leaves, stems, and pollen of the studied plant. The procedure was meticulously designed to ensure high-resolution imaging and accurate surface detail analysis. Samples of leaves, stems, and pollen were carefully prepared to preserve their structural integrity. Each sample was affixed onto double-sided adhesive carbon tape mounted on aluminum stubs, ensuring stability during the imaging process. To enhance the conductivity required for SEM analysis, the specimens were uniformly coated with a thin layer of gold using a pulse sputter coater (3.5 min of sputtering at optimized parameters). This coating minimized charging effects and improved image clarity. Micromorphological analysis was performed using an FEI INSPECT F50 SEM equipped with advanced imaging capabilities, operating at an acceleration voltage suitable for biological samples. The SEM provided high-resolution images, revealing intricate surface features and structural details. The leaves were examined for their epidermal patterns, stomatal arrangements, trichomes, and cuticular structures. The stems were analyzed for surface texture, arrangement of epidermal cells, and other distinguishing micromorphological features. Pollen samples were scrutinized for their shape, size, exine ornamentation, and apertural details, contributing to the taxonomic and ecological understanding of the plant. The SEM analysis yielded a comprehensive dataset of high-quality images and observations, which were systematically documented. These findings provided valuable insights into the micromorphology of the plant, contributing to its identification, characterization, and potential applications in botanical research.

### Statistical analysis

All experiments were performed in triplicate to ensure reliability. The Kruskal–Wallis test was utilized to assess statistical significance. Data analysis was carried out using SPSS software (IBM SPSS Statistics 20, IBM Corporation, Armonk, NY, USA), with a significance level established at *p* = 0.05. The IC_50_ values for the extracts are reported as mean ± standard deviation.

## Results

### Elemental analysis

The elemental composition analysis of the methanol extracts revealed significant variations in the concentration of various elements, as presented in Table 1. Using ICP-MS (inductively coupled plasma-mass spectrometry), 21 elements were investigated within the methanol extracts, including sodium (Na), magnesium (Mg), aluminum (Al), potassium (K), calcium (Ca), scandium (Sc), chromium (Cr), manganese (Mn), iron (Fe), cobalt (Co), zinc (Zn), arsenic (As), rubidium (Rb), strontium (Sr), cesium (Cs), barium (Ba), lanthanum (La), cerium (Ce), samarium (Sm), uranium (U), and selenium (Se). The results highlighted particularly high concentrations of Na and K. Specifically, Na levels ranged from 48,788.281 g/kg in sample HSR to 56,099.544 g/kg in sample HSAer, while K concentrations varied from 1,106.338 g/kg in sample HSFM to 21,472.225 g/kg in sample HSFr. Na and K, the most abundant elements detected, play crucial roles in maintaining cellular homeostasis, regulating osmotic balance, and supporting enzymatic functions. The notably high levels of Na and K across the samples may contribute to the physiological effects of these extracts, particularly in the context of their potential pharmacological applications.

**Table 1:** The composition of elemental analysis of methanol extracts

Elements	Major elements at g/kg concentrations							Trace elements at mg/kg concentrations						
	Na	K	Mg	Al	Ca	Fe	Zn	Ba	Sc	Cr	Mn	Co	As	Se
FM	48,896.458	1,106.338	845.108	1271.358	569.526	325.845	224.295	24.602	< 0.000	< 0.000	9.704	0.911	< 0.000	< 0.000
FAerM	50,216.192	12,425.066	2146.303	1215.455	156.573	240.786	254.847	19.873	< 0.000	< 0.000	10.759	0.180	< 0.000	< 0.000
AerM	56,099.544	2287.032	560.684	1230.661	155.066	244.348	154.426	18.445	< 0.000	< 0.000	3.301	< 0.000	< 0.000	< 0.000
FrM	51,951.392	21,472.225	3635.730	1247.622	175.750	231.231	154.356	30.529	< 0.000	< 0.000	12.874	0.412	< 0.000	< 0.000
RM	48,788.281	20,207.221	3701.154	1130.727	220.227	205.661	119.057	29.202	< 0.000	< 0.000	11.277	< 0.000	< 0.000	< 0.000

Elements	Ultratrace elements at µg/kg concentrations							
	Rb	Rb	Sr	Cs	La	Ce	Sm	U
FM	0.865	0.865	2.369	< 0.000	0.051	0.654	< 0.000	< 0.000
FAerM	4.456	4.456	1.022	< 0.000	0.032	0.449	< 0.000	< 0.000
AerM	1.183	1.183	1.308	< 0.000	< 0.000	0.440	< 0.000	< 0.000
FrM	7.561	7.561	1.107	< 0.000	< 0.000	0.361	< 0.000	< 0.000
RM	7.264	7.264	0.746	< 0.000	< 0.000	0.393	< 0.000	< 0.000

The definition of “trace element” as outlined in the IUPAC Compendium of Chemical Terminology, second edition, sets a threshold: “Any element having an average concentration of less than about 100 parts per million atoms or less than 100 μg/g.” However, advancements in analytical techniques have pushed detection capabilities further. In many fields, the upper boundary of the “trace” definition now falls short of the analytical precision achievable today. Consequently, terms like “ultra-trace analysis” have emerged to delineate this domain. Although there is no universally agreed-upon range for ultra-trace analysis, it generally refers to elements with mass fractions below 10 − 6 and 10 − 8 g/g (1 ppm and 10 ppb), respectively (Brown and Milton 2005)

*FM H. scabrum* flower methanol extract, *FAerM H. scabrum* aerial part with flower methanol extract, *AerM H. scabrum* aerial part methanol extract, *FrM H. scabrum* fruit methanol extract, *RM H. scabrum* root methanol extract

### Quantitative analysis of secondary metabolites

The quantitative analysis of 35 phenolic compounds in methanolic extracts, performed using LC–MS/MS, identified the presence of 22 compounds across the samples, with the detailed results provided in Table 2. Quinic acid emerged as the most abundant phenolic compound, particularly in the RM, FrM, and FAerM samples, with concentrations of 27,564.7251; 22,966.0034; and 17,135.8027 ng/mL, respectively. This suggests Quinic acid as a key constituent, potentially contributing to the bioactive and therapeutic properties of the plants. Additionally, high levels of other phenolic compounds were observed, including vanillic acid, which was present at 3,998.1174 ng/mL in the FM sample. Known for its antioxidant properties, vanillic acid may enhance the health benefits associated with these extracts. Chlorogenic acid was also found in significant quantities, ranging from 240.2110 to 13,940.5635 ng/mL, further highlighting its importance among the phenolic compounds detected. These findings underscore the potential bioactivity of these compounds and their contribution to the therapeutic value of the plant extracts (Table 3).

**Table 2:** Quantitative analysis of 35 different phenolic compounds via LC–MS/MS in methanol extracts

Compound	Final concentration (ng/mL)				
Samples	FM	FAerM	AerM	FrM	RM
Quinic acid	2105.0134	17,135.8027	3884.9826	22,966.0034	27,564.7251
Fumaric acid	0.0000	0.0000	0.0000	0.0000	0.0000
Gallic acid	4.4608	201.2532	424.7034	111.8155	142.6354
Pyrogallol	0.0000	0.0000	0.0000	0.0000	0.0000
Keracyanin chloride	8.4901	53.0493	16.4384	7.9750	38.8785
Cyanidin-3-O-glucoside	60.4766	550.9063	1637.2003	26.4620	27.3028
Chlorogenic acid	240.2110	8950.3765	13,940.5635	4840.8740	5100.2017
Catechin	0.0000	0.0000	168.8927	0.0000	0.0000
Peonidin-3-o-glucoside	0.0000	1.3183	3.5255	0.0000	8.3877
4-OH-benzoic acid	0.0000	0.0000	50.1961	0.0000	0.0000
Epicatechin	0.0000	0.0000	300.7311	0.0000	0.0000
Epigallocatechin gallate	91.0557	52.6803	20.9310	44.7204	40.7326
Caffeic acid	0.0000	0.0000	0.0000	0.0000	0.0000
Vanillic acid	3998.1174	2910.3339	139.4786	80.9191	2389.1645
Syringic acid	0.0000	6.2710	3.1477	0.0000	0.0000
Vitexin	0.0000	0.0000	0.0000	0.0000	0.0000
Naringin	0.0000	0.0000	22.1362	0.0000	0.0000
Ellagic acid	1189.9763	1121.8282	783.4836	341.9689	213.6145
Hesperidin	0.0000	0.0000	1.6207	0.0000	0.0000
p-Coumaric acid	0.0000	0.0000	0.0000	0.0000	0.0000
Sinapic acid	0.0000	0.0000	0.0000	0.0000	0.0000
Taxifolin	0.0000	0.0000	0.0000	0.0000	0.0000
Ferulic acid	0.0000	1166.3867	0.0000	614.4054	605.5475
Rosmarinic acid	17.4477	188.8948	177.9801	36.5613	19.1125
Vanillin	46.6138	45.2531	43.5487	43.2419	43.3103
Myricetin	0.0000	1346.8703	4685.8370	162.6340	12.0174
Resveratrol	1.9812	2.1532	2.5640	2.3348	2.2932
Luteolin	0.0000	3.8677	70.4756	0.0000	0.0000
Quercetin	0.0000	907.1214	2378.9054	54.0232	0.0000
Apigenin	0.0000	0.0000	0.0000	0.0000	0.0000
Naringenin	0.0000	0.0000	0.0000	0.0000	0.0000
Isorhamnetin	0.0000	0.0000	0.0000	0.0000	0.0000
Chrysin	0.0000	0.0000	0.0000	0.0000	0.0000
Galangin	0.0000	0.0000	0.0000	0.0000	0.0000
Curcumin	0.0000	0.0000	0.0000	ND	0.0000

*FM H. scabrum* flower methanol extract, *FAerM H. scabrum* aerial part with flower methanol extract, *AerM H. scabrum* aerial part methanol extract, *FrM H. scabrum* fruit methanol extract, *RM H. scabrum* root methanol extract, *ND* not detected

**Table 3:** Amino acid compositions of methanol extract

Amino Acids	Concentration (nmol/mL)				
	FM	FAerM	AerM	FrM	RM
Tryptophan	30.5208	17.1109	18.1138	31.0448	18.4001
Taurine	22.5481	30.5855	36.3069	14.2695	31.3172
Phenylalanine	0.0000	0.0000	0.0000	0.0000	0.0000
Tyrosine	30.3069	49.5512	36.3909	33.0442	48.0416
Leucine	13.9041	41.9223	12.0918	20.8233	16.7641
Isoleucine	63.0824	74.6013	12.2569	95.6012	89.0485
Methionine	0.0000	0.0000	70.3891	0.0000	0.0000
3-Aminoisobutyric acid	0.0000	0.0000	0.0000	0.0000	0.0000
Gamma-aminobutyric acid	0.0000	0.0000	0.0000	0.0000	0.0000
2-Aminoadipic acid	10.2316	9.9316	0.0000	17.3767	17.5318
Norvaline	0.0000	0.0000	0.0000	0.0120	0.1978
Glutamic acid	48.0792	0.0000	0.0000	8.4176	0.0000
Beta-alanine	4.7861	58.7428	25.0039	55.3011	14.7813
Ethanolamine	0.0000	0.0000	57.6277	0.0000	51.5732
Aspartic acid	27.3372	6.2744	0.0000	42.0896	0.0000
Valine	0.0000	46.6024	1.5584	0.0000	31.3711
2-Aminobutyric acid	0.0000	0.0000	0.0000	0.0000	0.0000
Threonine	0.0000	0.0000	0.0000	0.0000	0.0000
Serine	0.0000	0.0000	0.0000	0.0000	0.0000
Alanine	58.8550	124.3387	91.5595	174.9001	164.4457
Glycin	0.0000	0.0000	23.6206	13.7660	301.0810
Trans-4-hydroxyproline	15.8029	13.8502	13.2927	0.0000	26.5692
Proline	0.0000	25.7627	494.0482	17.4257	13.6123
Glutamine	0.0000	117.2463	25.7167	394.6668	0.0000
Asparagine	20.4643	0.0000	0.0000	0.0000	473.3873
Sarcosine	0.0000	0.0000	0.0000	0.0000	0.0000
Homocitrulline	0.0000	0.0000	0.0000	0.0000	0.0000
Ortho-phosphorylethanolamine	0.0000	0.0000	0.0000	0.0000	0.0000
Citrulline	0.0000	0.0000	0.0000	0.0000	0.0000
Homocystine	0.0000	0.0000	0.0000	0.0000	0.0000
Ortho-phosphoserine	0.0000	0.0000	0.0000	0.0000	0.0000
Cystine	0.0000	0.0000	0.0000	0.0000	0.0000
Argininosuccinic acid	0.0000	0.0000	0.0000	0.0000	0.0000
Cystathionine	0.0000	34.8435	39.4918	0.0000	0.0000
Arginine	25.8044	0.0000	0.0000	26.5625	0.0000
5-Hydroxylysine	0.0000	0.0000	0.0000	0.0000	30.2816
Histidine	0.0000	0.0000	0.0000	0.0000	48.8627
Carnosine	0.0000	0.0000	0.0000	0.0000	0.0000
Ornithine	0.0000	0.0000	37.0720	0.0000	0.0000
Lysine	33.5216	0.0000	0.0000	46.3623	0.0000
1-Methylhistidine	0.0000	49.1176	0.0000	0.0000	0.0000
3-Methylhistidine	0.0000	0.0000	0.0000	0.0000	0.0000
Anserine	0.0000	0.0000	0.0000	0.0000	0.0000

*FM H. scabrum* flower methanol extract, *FAerM H. scabrum* aerial part with flower methanol extract, *AerM H. scabrum* aerial part methanol extract, *FrM H. scabrum* fruit methanol extract, *RM H. scabrum* root methanol extract

### Cytotoxicity analyses

**Fig. 1:**
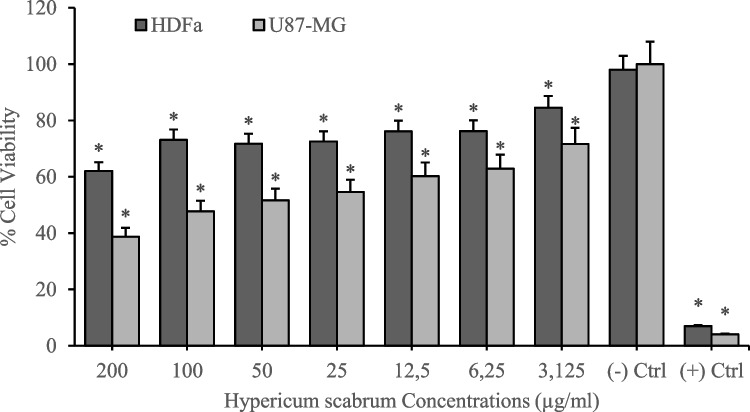
Cytotoxicity and anticancer analyses of *H. scabrum* L. dH_2_O isolate on human fibroblast (HDFa) cell line and human glioblastoma (U87MG) cell culture for 24 h of application

**Fig. 2:**
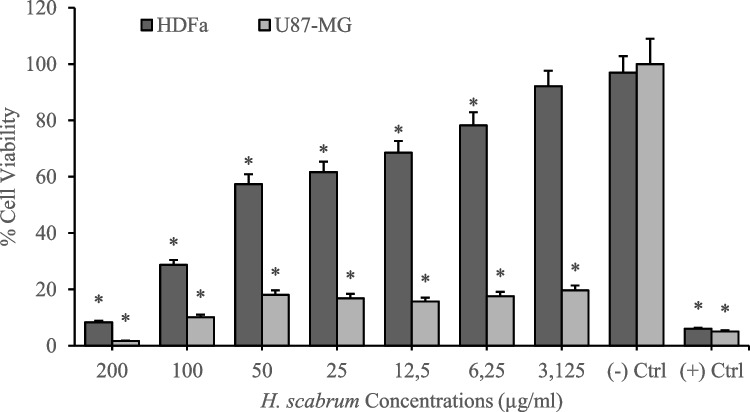
Cytotoxicity and anticancer analyses of *H. scabrum* L. CH_2_Cl_2_ isolate on human fibroblast (HDFa) cell line and human glioblastoma (U87MG) cell culture for 24 h of application

**Fig. 3:**
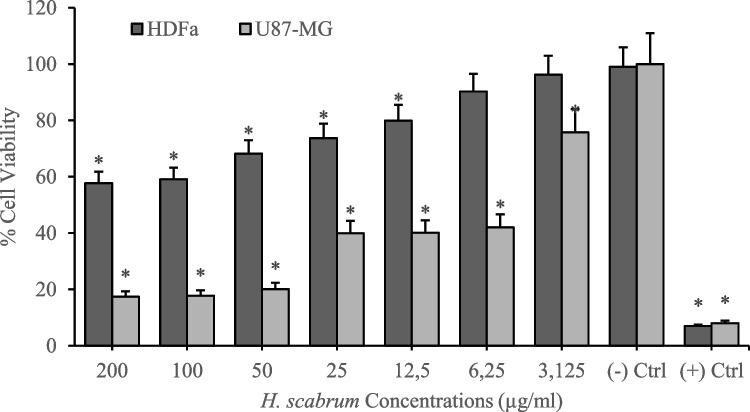
Cytotoxicity and anticancer analyses of *H. scabrum* L. EtOAc isolate on human fibroblast (HDFa) cell line and human glioblastoma (U87MG) cell culture for 24 h of application

**Fig. 4:**
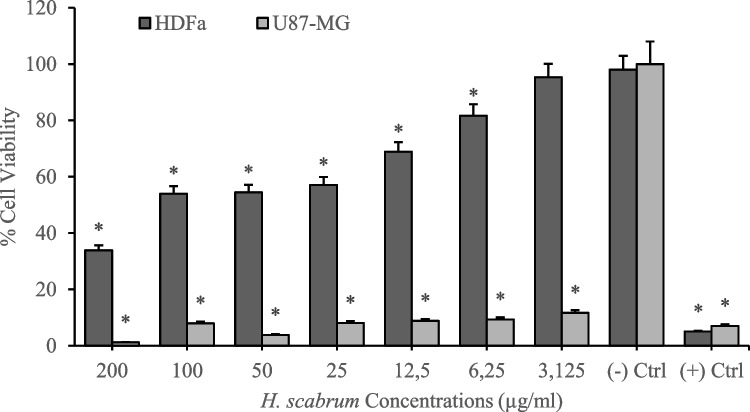
Cytotoxicity and anticancer analyses of *H. scabrum* L. MeOH isolate on human fibroblast (HDFa) cell line and human glioblastoma (U87MG) cell culture for 24 h of application

**Fig. 5:**
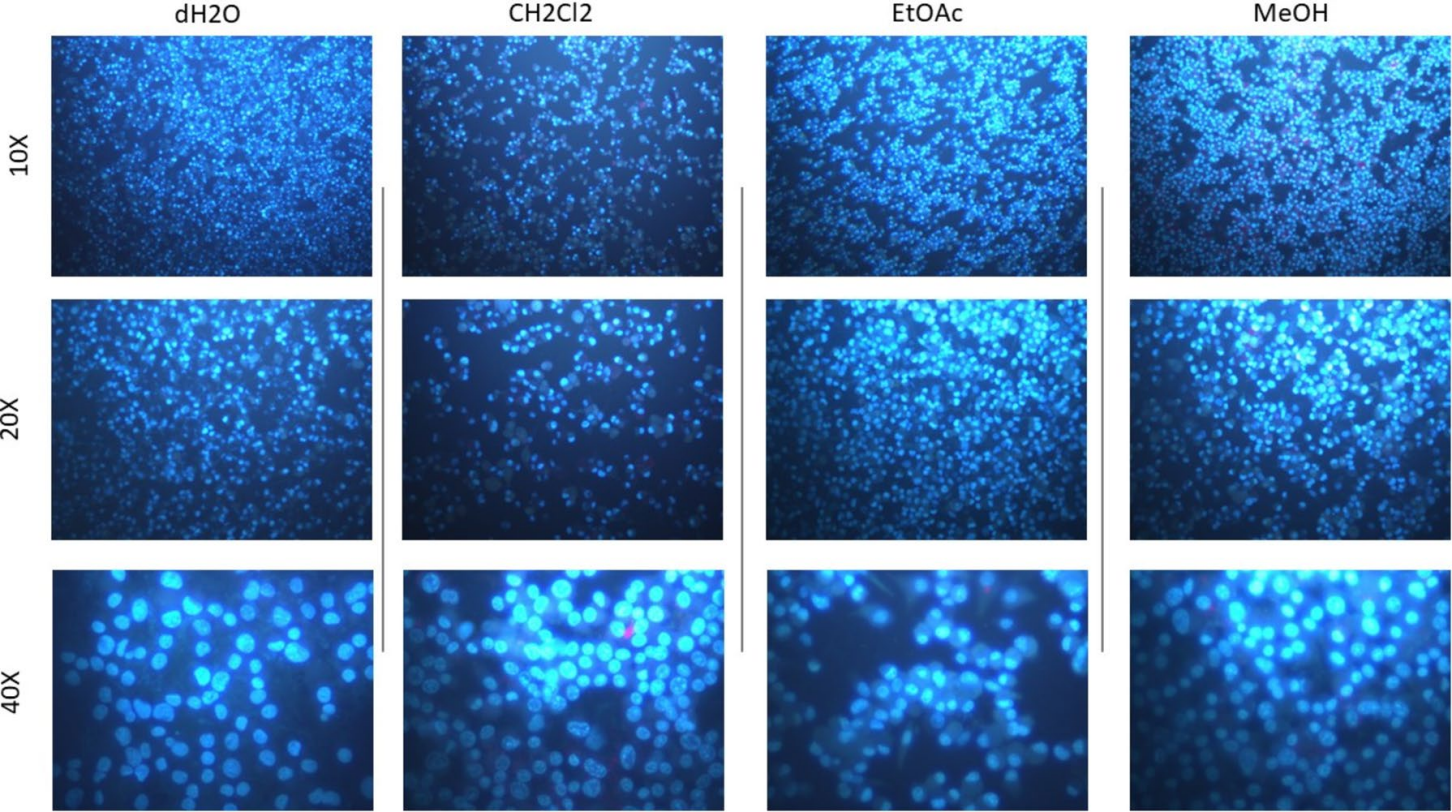
Nuclear abnormality analysis of *H. scabrum* L. isolates (200 μg/mL) using Hoechst 33,258 fluorescent nuclear staining method

Cytotoxicity analyses of *H. scabrum* isolates on the human fibroblast cell line (HDFa) were investigated by using MTT cell viability assay after 24 h of treatments. According to the results, different *H. scabrum* isolates (dH_2_O, CH_2_Cl_2_, EtOAc, and MeOH) exhibited comparatively low toxicity on the HDFa cell line. Analyses put forth that 200 µg/mL of concentration of dH_2_O and EtOAc isolates resulted in nearly 40% cytotoxicity. On the other hand, CH_2_Cl_2_ and MeOH extract showed higher cytotoxicity at 200 µg/mL of concentration which led to an average of 70% cytotoxicity in the HDFa cell culture (Figs. 1, 2, 3, 4, and 5). Besides, anticancer analyses of the extracts showed higher cytotoxicity on the human glioblastoma (U87MG) cell culture compared to the healthy human fibroblast (HDFa) cell line. The most competent anticarcinogens were observed as *H. scabrum* CH_2_Cl_2_ and MeOH isolates on the U87MG cell cultures with 98% cytotoxicity at 200 µg/mL of concentration after 24 h of applications (Figs. 1, 2, 3, 4, and 5). The findings, revealing comparatively low toxicity of dH2O and EtOAc isolates on the HDFa cell line, align with the broader literature indicating that the selectivity of plant extracts toward cancerous cells is an essential criterion for anticancer drug development.

Following the cytotoxic evaluations, the investigation progressed to genotoxic analyses employing Hoechst 33,258 staining, a technique that illuminates nuclear aberrations including micronuclei, lobulated, and notched nuclei. These irregularities serve as vital markers for DNA harm and genomic instability, highlighting the mutagenic or carcinogenic dangers linked to the substances being tested. The study scrutinized genotoxic effects at an elevated concentration of 200 µg/mL to assess the severity of DNA and chromosome distortions in cells treated with *H. scabrum* extracts. Results indicated no marked increase in nuclear anomalies in the treated groups compared to the untreated control group, which had not been exposed to the *H. scabrum* extracts. This outcome implies that, at the evaluated concentration, the *H. scabrum* extracts did not cause DNA or chromosomal damage significantly beyond the normal range of nuclear abnormalities found in control cells (Fig. 5 and Table 4).

**Table 4:** Nuclear abnormalities (NA) in HDFa cell line against *H. scabrum* L. isolates applications on human fibroblast (HDFa) cell culture for 24 h (200 μg/mL)

Treatments	Nuclear abnormalities (NA)			
Isolates	Total MN	Total lobbed	Total notched	Mean NA/1000 cells ± SD
(-) Ctrl	5	2	5	0.012 ± 0.001^a^
dH_2_O	3	4	7	0.014 ± 0.002^a^
CH_2_Cl_2_	4	4	5	0.013 ± 0.001^a^
EtOAc	3	5	5	0.013 ± 0.003^a^
MeOH	5	3	3	0.011 ± 0.002^a^

To evaluate the anticancer potential of *H. scabrum* isolates, the selectivity indexes (SI) were calculated by taking the IC_50_ value for the HDFa cell line and dividing it by the IC_50_ value for the U87MG cell line. The IC_50_ value refers to the concentration of the substance required to inhibit cell viability by 50%. A higher SI value indicates a greater specificity of the extract toward cancer cells, which is a desirable attribute for anticancer treatments, as it suggests that the substance can effectively target cancer cells while minimizing damage to healthy cells. The CH_2_Cl_2_ and MeOH extracts demonstrate exceedingly high selectivity toward U87MG cells with SI values of ≥ 200 (Table 5). *H. scabrum* CH_2_Cl_2_ and MeOH extracts display a high degree of selectivity toward cancer cells, suggesting their promise as candidates for anticancer drug development. The dH2O extract’s low selectivity may limit its therapeutic use, whereas the EtOAc extract presents a potential for further investigation.

**Table 5:** Selectivity indexes (SI) of *H. scabrum* L. isolates calculated by using IC_50_ values on HDFa healthy cell line compared to U87MG cancer cell culture

Treatments	U87MG (IC_50_)	HDFa (IC_50_)	Selectivity index (SI)
dH_2_O	87.4443	295.8417	3.3832
CH_2_Cl_2_	≤ 0.001	77.5888	≥ 200
EtOAc	5.263	277.2233	52.674
MeOH	≤ 0.001	116.8834	≥ 200

### α-Glucosidase and α-amylase inhibition assays

The Table 6 provided insights into the antidiabetic activity of various extracts and essential oils, showcasing IC_50_ values for *α*-glucosidase.

**Table 6:** In vitro antidiabetic activity of extracts and essential oils of *H. scabrum*

Samples	α-Glucosidase inhibition IC_50_ values (µg/mL)
Roots	
Methanol	1076
*n*-Hexane	14
Dichloromethane	452
Ethyl acetate	88
*n*-Butanol	15
Aqueous residue	10
Fruits	
Methanol	854
*n*-Hexane	446
Dichloromethane	330
Ethyl acetate	11
*n*-Butanol	7
Aqueous residue	14
Essential oil	ND
Flowering aerial parts	
Methanol	259
*n*-Hexane	528
Dichloromethane	762
Ethyl acetate	23
*n*-Butanol	7
Aqueous residue	1224
Aerial parts	
Methanol	< 1
*n*-Hexane	947
Dichloromethane	239
Ethyl acetate	79
*n*-Butanol	7
Aqueous residue	309
Flowers	
Methanol	4082
*n*-Hexane	806
Dichloromethane	237
Ethyl acetate	6
*n*-Butanol	27
Aqueous residue	1063
Essential oil	11,364
Acarbose*	3230

*ND* not determined

^*^Positive control for antidiabetic activity

The provided table highlights the *α*-glucosidase inhibition activity of various extracts and essential oils from *Hypericum scabrum*, presented as IC_50_ values. These values represent the concentration of each extract required to inhibit 50% of *α*-glucosidase activity. Acarbose, a well-known antidiabetic drug, is used as a positive control, with an IC_50_ value of 3230 µg/mL.

When comparing the extracts to acarbose, several stand out with notably lower IC_50_ values, indicating higher potency. For example, the methanol extract of the aerial parts (IC_50_ < 1 µg/mL), ethyl acetate extracts of fruits (11 µg/mL) and flowers (6 µg/mL), and the *n*-butanol extracts from various parts (ranging from 7 µg/mL to 27 µg/mL) all exhibited significantly stronger *α*-glucosidase inhibition compared to acarbose. The aqueous residue of roots and fruits also showed strong activity, with IC_50_ values of 10 µg/mL and 14 µg/mL, respectively.

Interestingly, the essential oils generally demonstrated weak activity, with values exceeding 11,364 µg/mL for the flowers, making them much less effective than acarbose. In contrast, the *n*-hexane and dichloromethane extracts from different parts showed varying levels of inhibition, with some extracts performing better than acarbose, such as the *n*-hexane extract of roots (14 µg/mL) and dichloromethane extract of flowers (237 µg/mL), though these values were still higher than other more potent extracts.

Overall, this data suggests that specific extracts, especially those from methanol, ethyl acetate, and *n*-butanol, have much stronger antidiabetic potential compared to the standard drug acarbose. These findings indicate the potential of *H. scabrum* extracts for further exploration as natural *α*-glucosidase inhibitors.

### Acetylcholinesterase (AChE) and butyrylcholinesterase (BChE) inhibition assays

The Table 7 provided insights into the anticholinesterase activities of various extracts and essential oils, showcasing inhibition (%) AChE and BChE.

**Table 7:** In vitro anticholinesterase activities of extracts and essential oils of *H. scabrum*

Samples	Acetylcholinesterase inhibition (%) (100 µg/mL) (mean ± std)	Butyrylcholinesterase inhibition (%) (1000 µg/mL) (mean ± std)
Roots		
Methanol	10.58 ± 1.60	11.21 ± 4.12
*n*-Hexane	11.71 ± 7.60	34.86 ± 3.94
Dichloromethane	14.53 ± 3.65	31.46 ± 0.54
Ethyl acetate	6.95 ± 4.13	19.49 ± 4.14
*n*-Butanol	13.04 ± 6.61	22.44 ± 3.02
Aqueous residue	11.79 ± 1.05	23.22 ± 5.89
Fruits		
Methanol	18.55 ± 7.63	10.48 ± 3.81
*n*-Hexane	14.11 ± 4.68	28.37 ± 0.72
Dichloromethane	10.92 ± 1.97	20.77 ± 6.34
Ethyl acetate	15.30 ± 4.64	ND
*n*-Butanol	15.96 ± 3.14	10.64 ± 2.81
Aqueous residue	12.78 ± 4.42	20.67 ± 7.59
Essential oil	16.92 ± 2.25	11.33 ± 4.85
Flowering aerial parts		
Methanol	2.49 ± 1.90	26.10 ± 2.00
*n*-Hexane	4.34 ± 4.53	29.60 ± 5.91
Dichloromethane	11.99 ± 2.65	27.24 ± 4.37
Ethyl acetate	0.78 ± 3.69	ND
*n*-Butanol	17.82 ± 6.88	6.45 ± 2.51
Aqueous residue	10.18 ± 3.28	12.41 ± 3.35
Aerial parts		
Methanol	15.72 ± 4.99	15.63 ± 0.78
*n*-Hexane	10.82 ± 5.11	38.32 ± 0.73
Dichloromethane	12.59 ± 4.40	34.23 ± 2.30
Ethyl acetate	12.13 ± 4.62	ND
*n*-Butanol	10.34 ± 4.92	7.13 ± 1.47
Aqueous residue	12.24 ± 1.22	13.83 ± 4.54
Flowers		
Methanol	3.55 ± 3.45	17.34 ± 1.83
*n*-Hexane	ND	27.36 ± 0.85
Dichloromethane	7.41 ± 4.75	30.64 ± 1.55
Ethyl acetate	7.11 ± 5.39	ND
*n*-Butanol	7.36 ± 7.40	ND
Aqueous residue	ND	14.08 ± 2.10
Essential oil	21.62 ± 4.49	79.95 ± 2.35
Donepezil*	97.59 ± 1.13	100 ± 1.27

*ND* not determined

^*^Positive control for anticholinesterase activities

The provided table presents the in vitro anticholinesterase activities of various extracts and essential oils from *H. scabrum*, comparing their inhibition percentages for acetylcholinesterase (AChE) and butyrylcholinesterase (BChE) enzymes at concentrations of 100 µg/mL and 1000 µg/mL, respectively. Donepezil, a well-known cholinesterase inhibitor used in treating Alzheimer’s disease, serves as the positive control, exhibiting nearly complete inhibition of both enzymes (97.59% for AChE and 100% for BChE).

The results indicate that most of the tested extracts and essential oils exhibit relatively low inhibition of AChE compared to donepezil. The highest AChE inhibition was observed in the essential oil from flowers (21.62%), followed by *n*-butanol from flowering aerial parts (17.82%), fruits (15.96%), and aerial parts methanol extract (15.72%). However, these values are much lower than donepezil’s 97.59%, indicating that none of the extracts are particularly potent inhibitors of AChE at the tested concentrations.

For BChE inhibition, certain extracts showed more promising results, though still not comparable to donepezil. The essential oil from flowers exhibited the highest inhibition (79.95%), making it the most potent BChE inhibitor among the tested samples. Other notable BChE inhibitors included the *n*-hexane extract from aerial parts (38.32%), dichloromethane extract from the same source (34.23%), and *n*-hexane extract from roots (34.86%). Despite these moderate levels of inhibition, they are still far below the 100% inhibition seen with donepezil.

In terms of overall trends, it is clear that the essential oils generally performed better as BChE inhibitors than as AChE inhibitors. On the other hand, most of the extracts, regardless of the plant part or solvent used, did not exhibit significant anticholinesterase activity, particularly against AChE. While the essential oil from flowers shows potential for BChE inhibition, the other extracts are less potent.

### Morphological analysis

#### *Hypericum scabrum* L., Cent. Pl. I: 25 (1755)

**Fig. 6:**
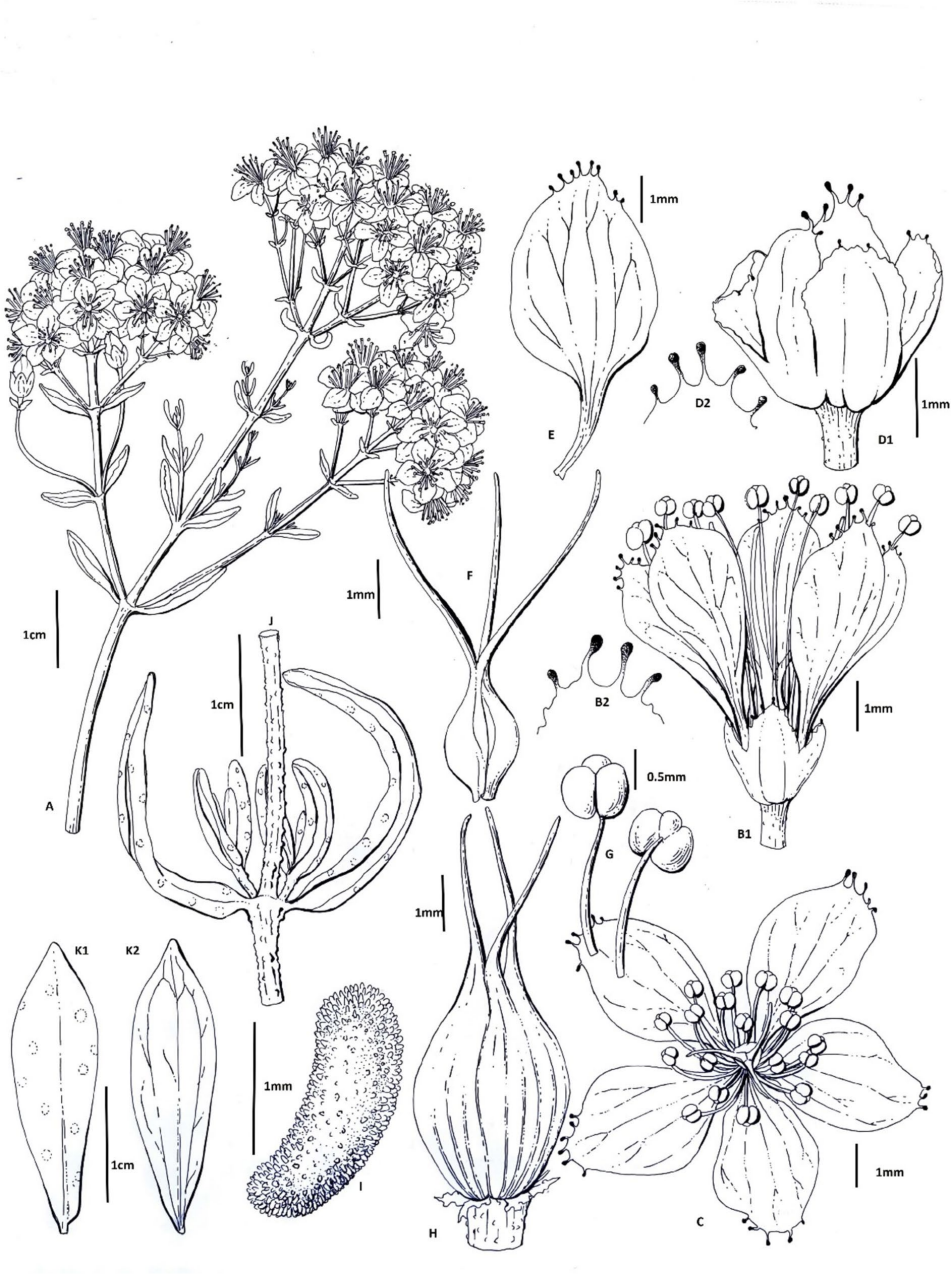
General appearance (A), flowers (B, C), calyx (D), petal (E), pistil (F), capsıl (G), seed (H), cross-section from stem (I), leaf upper surface (J1), leaf lower surface (J2)

Stems 20–50 cm, scabrid with unbranched emergences. Leaves 5–30 mm, oblong, oblong-elliptic to lanceolate or linear, glabrous at lower surface, glandular at upper surface. Inflorescence corymbose, with more than 15 flowers. Petals 7–8 mm long, irregularly glandular denticulate to ciliate at apex, yellow; glands dark violet. Pistil equal to or slightly shorter than stamens. Stamens longer than perianth; anthers 0.5 mm in length, yellow. Sepals oblong to ovate, united up to c. 1/3–1/2 of calyx, subacute and irregularly glandular denticulate to ciliate at apex, green. Capsule 5–8 mm, ovoid, ribbed. Seeds 1.5–2 mm in length, reniform, brown, covered with transparent scales. General appearance (A), flowers (B, C), calyx (D), petal (E), pistil (F), capsıl (G), seed (H), cross-section from stem (I), leaf upper surface (J1), and leaf lower surface (J2) are presented in Fig. 6.

### Microscopic analysis

#### Leaf micromorphology

**Fig. 7:**
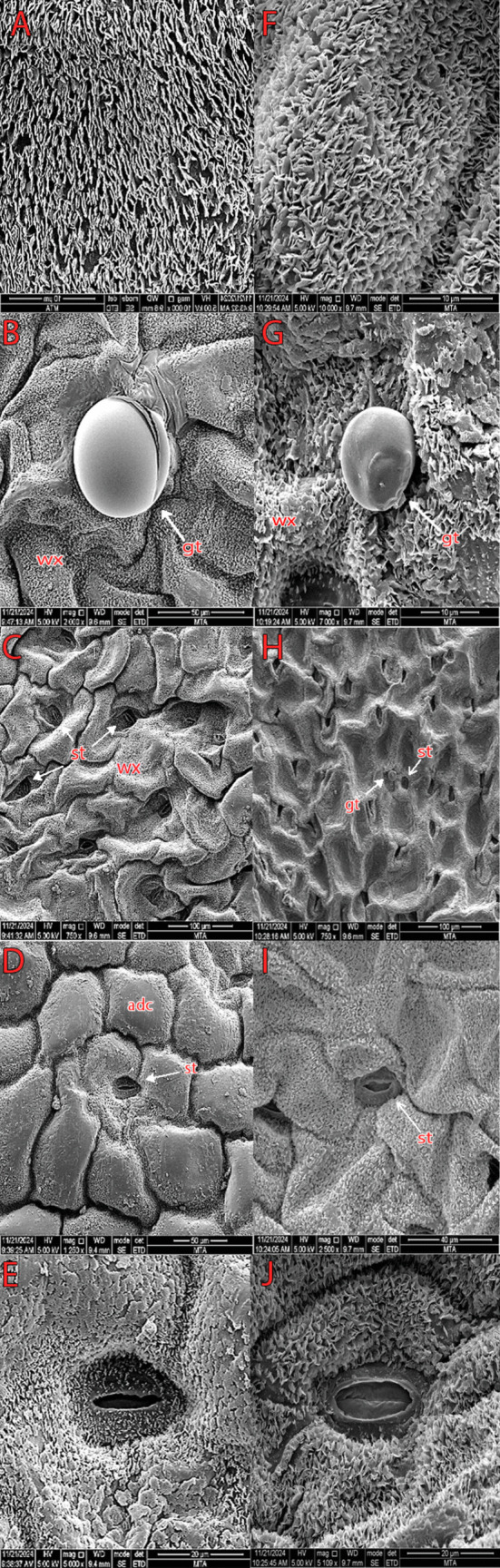
Scanning electron microscope (SEM) micromorphology of leaves on adaxial surfaces of lamina. **A** Cuticular ornamentation. **B** Glandular trichome. **C**–**E** Distribution of stomata and stomata type and abaxial surfaces of lamina. **F** Cuticular ornamentation. **G** Glandular trichome. **H**–**J** Distribution of stomata and stomata type. Abbreviations: wx, cuticular wax; adc, adaxial epidermis cell; st, stoma; gt, glandular trichome

SEM shows the epidermal morphology of the adaxial and abaxial surfaces to be rich in cuticular ornamentation. The epidermal cells present “flake” ornamentation arranged vertically, very densely on the body of the epidermal cells. On both leaf surfaces, the veins are characterized by numerous rows of rectangular cells, more or less elongated and paired, but offset by about half a cell. It has amphistomatic leaves. Stomata are present on both adaxial and abaxial sides. Number of stomata per 100 µm^2^ is 2–3 on both adaxial and abaxial sides. The guard cell shape is *Amaryllis* type. Stomata are anomocytic on the adaxial surface and anisocytic on the abaxial surface. Length of stomata ranges from 19.4 (15–23) μm, stomata width 20.1 (16–23) μm, stomatal pore length 14 (13–15) μm, and stomatal pore width 2 (1.7–3.6) μm on the abaxial surface and stomata length 22.4 (19–25) μm, stomata width 21 (16–23) μm, stomatal pore length 14.2 (13–15.4) μm, and stomatal pore width 2 (1.7 –3.5) μm on the adaxial surface. There is ornamentation in the guard cells. It has secretory trichomes on both sides. Number of secretory trichomes per 100 µm^2^ is 0–1 on both adaxial and abaxial sides (Fig. 7).

#### Stem micromorphology

**Fig. 8:**
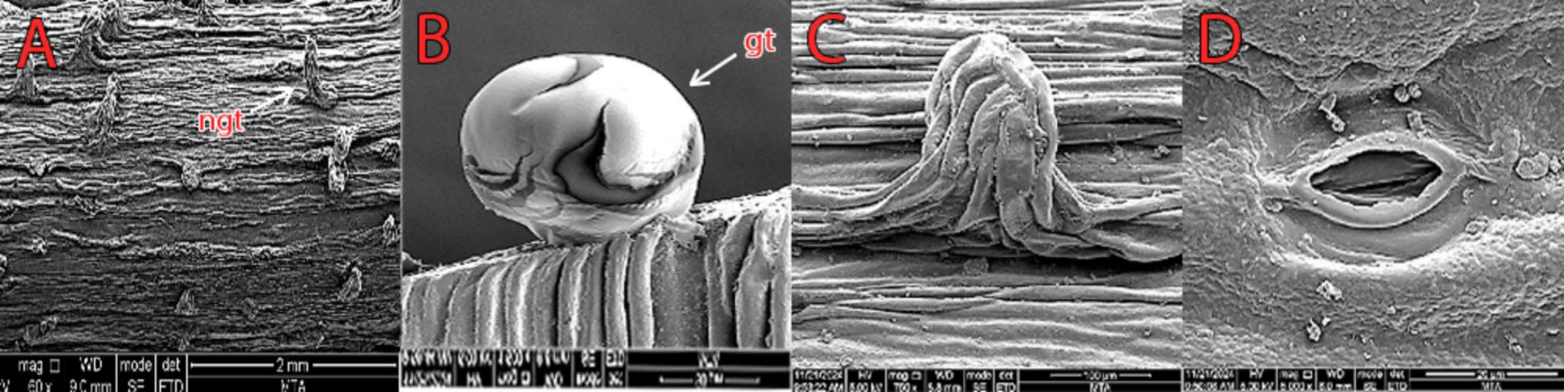
Scanning electron microscope (SEM) micromorphology of stem. **A** Striate cuticular ornamentation. **B** Glandular trichome. **C** Nonglandular trichome. **D** Stomata type. Abbreviations: ngt, nonglandular trichome; gt, glandular trichome

Striate cuticular ornaments were observed on the epidermis cells. Stomata are present, and the number of stomata per 100 µm^2^ is 0–1. The guard cell shape is *Amaryllis* type, and the stomata are paracytic. Length of stomata ranges from 17 (15–19) μm, stomata width 10.1 (9–11.2) μm, stomatal pore length 7 (6–8) μm, and stomatal pore width 2.3 (1.5–4) μm. There is no ornamentation in the guard cells. It has secretory trichomes, and the number of secretory trichomes per 100 µm^2^ is 0–1 (Fig. 8).

#### Pollen micromorphology

**Fig. 9:**
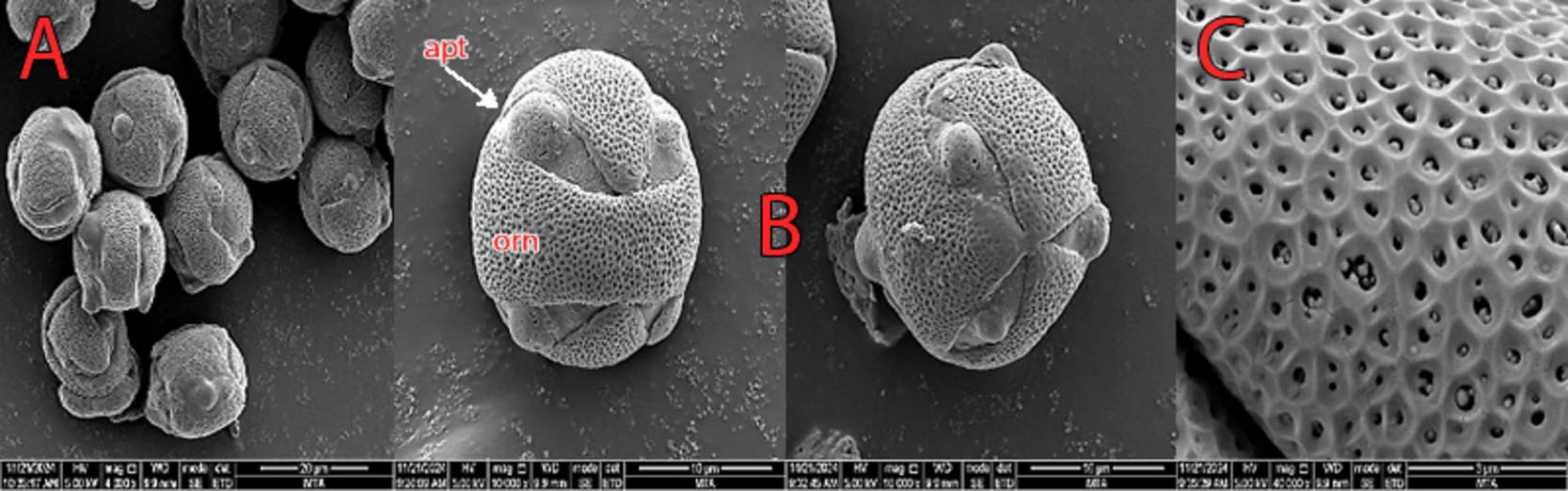
Scanning electron microscope (SEM) micromorphology of pollen. **A** Pollen shape. **B** Aperture types. **C** Exine pattern. Abbreviations: apt, aperture; orn, ornamentation

The pollen grains are monad, subisopolar, and spheroidal or oblate-spheroidal in shape. Size varies, with the polar axis ranging from 17 to 19.6 μm and the equatorial axis from 17.3 to 20 μm. Aperture types are 4–5–6 syncolporate. Exine sculpturing is microreticulate including small lumen/thick muri (Fig. 9).

## Discussion

### Elemental analysis

The mineral content of raw extracts from the leaf and flower parts of *H. scabrum* was analyzed using ICP-OES. The concentrations of toxic (Cd, Pb, Cr, Ba, As, Al, Ni), micro (Cu, Fe, Mn, B, Co, Se, Zn), and macro minerals (Ca, P, Na, Si, Mg) were determined. The mineral levels in both parts were similar. Vegetation near roadsides tends to diminish, and heavy metal concentrations are higher in residential and industrial areas. Element accumulation varies by plant part, with concentrations of Mn, Ni, Cr, Cu, Cd, Pb, and Zn generally aligning with reported values (Dastan 2023).

Na is an essential element in the body, supporting various physiological functions. As the body cannot produce it, sodium must be obtained through the diet, found in both animal and plant-based foods. It plays a key role in maintaining proper bodily functions. K is crucial for biological functions, yet its role in life’s origins is puzzling due to its lower abundance in seawater compared to Na. Its necessity for protein synthesis and metabolic pathways suggests it may have been specifically selected, or its role may result from life emerging in potassium-rich environments by chance (Kodintsev et al., 2022; Danchin and Nikel 2019).

Additionally, several heavy metals, such as Al, Mn, Fe, Co, Zn, Rb, Sr, Ba, La, and Ce, were detected in the extracts. While some of these metals, such as Fe and Zn, are essential micronutrients involved in redox reactions and enzymatic processes, others like Al and certain rare earth elements (e.g., La, Ce) raise concerns due to their potential toxicity at elevated levels. Trace elements are essential minerals present in living organisms at microgram levels per gram of body weight. Key trace elements crucial for human health include iodine, iron, zinc, cobalt (as vitamin B12), copper, selenium, boron, and chromium. They are integral to various physiological functions, such as acting as enzyme components, enabling metabolic energy transfer, transporting oxygen, and regulating biological processes by interacting with cell membranes and influencing gene expression (Nielsen 2003).

However, the concentrations detected in the current study appear within a range that warrants further exploration into their bioavailability and accumulation, which may be influenced by environmental factors, such as soil composition, water sources, and atmospheric conditions. These external factors could explain the observed variability in elemental concentrations across different samples. Moreover, the detection of elements like scandium (Sc), rubidium (Rb), and cesium (Cs), though in trace amounts, points to the potential environmental interactions affecting the plants from which these extracts were derived. Sc, in particular, is not commonly reported in plant-based studies, and its presence could provide novel insights into the elemental assimilation capabilities of these species. The variability in elemental concentrations may also reflect physiological differences in the plant parts used for extraction or regional growing conditions. This warrants further studies to correlate these variations with biological activity, as the elemental profile could significantly influence the extract’s efficacy and safety. For instance, the presence of higher metal concentrations may alter the bioavailability of bioactive compounds or interact synergistically or antagonistically with other elements in biological systems.

These findings suggest a notable presence of essential and non-essential elements, providing valuable insights into the chemical profile of the extracts, which could be crucial for understanding their biological and pharmacological effects. The variability in element concentrations also indicates potential environmental or physiological factors influencing the elemental uptake in the plant material, offering further avenues for detailed investigation.

### Quantitative analysis of secondary metabolites

The genus *Hypericum* is rich in biologically active metabolites like hyperforin, hypericins, and phenolics. This study examined hyperforin, hypericin, pseudohypericin, chlorogenic acid, and flavonoids (rutin, hyperoside, apigenin-7-O-glucoside, kaempferol, quercitrin, quercetin, amentoflavone) in two Turkish species: *H. scabrum* and *H. bupleuroides*. Aerial parts from 30 plants were collected at full bloom; dissected into floral, leaf, and stem tissues; and analyzed for secondary metabolites by HPLC. All metabolites were found in both species, though hyperforin was absent in *H. scabrum* (Ayan et al. 2009). LC–MS/MS analysis showed that *H. scabrum* is rich in phenolic compounds, with 29 phytochemicals identified both qualitatively and quantitatively from 56 standards. Extracts varied based on solvent polarity. The methanol extract (ME) contained 24 phenolics, while the water extract (WE) had 20. Major phenolics in ME include quinic acid (31.24 mg/g), quercitrin (22.01 mg/g), and isoquercitrin (17.67 mg/g). In WE, quinic acid (103.95 mg/g) was the most abundant, followed by protocatechuic acid (6.42 mg/g). Other notable compounds, such as fumaric acid, caffeic acid, and rutin, were also found in medium to high amounts (Altay et al. 2022).

Quinic acid is a cyclic polyol that has shown anticancer effects against various types of cancer (Inbathamizh and Padmini 2013). Quinic acid, found in fruits like apples, berries, and coffee beans, is a nutraceutical used in the creation of important pharmaceutical compounds. While platinum-based drugs like cisplatin are widely used in cancer treatment, including oral cancer, their side effects and resistance issues have prompted research into phytotherapy as a potential source of safer, effective anticancer compounds (Singh et al. 2018).

### Amino acid measurement analysis by LC–MS/MS

Hyperforin and adhyperforin contribute to the antidepressant effects of *H. perforatum*. In shoot cultures, branched-chain amino acids were shown to be involved in their biosynthesis. Supplementing with 2 mM unlabelled L-isoleucine increased adhyperforin by 3.7 times, while 3 mM L-threonine boosted it by 2.0 times. L-valine had no effect on hyperforin production (Karppinen et al. 2007). The flowering tops of *H. hirsutum*, *H. montanum*, *H. perforatum* subsp. *angustifolium*, *H. perforatum* subsp. *perforatum*, and *H. perforatum* subsp. *veronense*, collected from various Croatian locations, were analyzed for flavonoid, phenolic acid, and amino acid composition. Thin-layer chromatography (TLC) identified ten flavonoids, three phenolic acids, and sixteen amino acids. Flavonoid content was highest in the three subspecies of *H. perforatum*, with *H. perforatum* subsp. *perforatum* showing the richest composition in flavonoids, phenolic acids, and amino acids (Maleš et al. 2004).

The methanolic extracts underwent a comprehensive quantitative analysis using LC–MS/MS, targeting the concentrations of 43 distinct amino acids. Out of these, 26 amino acids were successfully identified across various samples, with detailed results presented in Table 3. Proline was the most abundant amino acid detected, with concentrations reaching up to 494.0482 nmol/mL in the AerM sample. Proline’s high concentration is notable given its crucial role in stress tolerance, osmoprotection, and protein synthesis, indicating its significant involvement in the physiological and metabolic activities of the plant. Asparagine, another amino acid essential for nitrogen assimilation and protein biosynthesis, was also present in large quantities, particularly in sample RM, where its concentration reached 473.3873 nmol/mL. This suggests a substantial presence of nitrogen-containing compounds in the plant, which may contribute to its nutritional value and metabolic characteristics. In addition to proline and asparagine, significant levels of glutamine were detected, particularly in the FrM sample, where its concentration reached 394.6668 nmol/mL. Glutamine, known for its roles in nitrogen transport, stress response, and regulation of metabolic pathways, further underscores the plant’s capacity to respond to environmental stressors and maintain metabolic homeostasis. Furthermore, alanine was found in high concentrations in both the FM and FAerM samples, with values of 58.8550 nmol/mL and 124.3387 nmol/mL, respectively. Alanine plays a pivotal role in glucose metabolism, serving as a substrate in gluconeogenesis, and its presence reflects the plant’s involvement in carbohydrate metabolic pathways. Sarcosine, another amino acid involved in methylation and associated with muscle function and energy metabolism, was also detected, though in lower quantities, pointing towards the plant’s complex metabolic network.

This detailed amino acid profiling not only sheds light on the metabolic pathways active in the plant but also hints at its potential nutritional and therapeutic applications. The presence of these amino acids, particularly those involved in stress responses and metabolic regulation, highlights the bioactive potential of the methanolic extracts.

Cancer remains a major global health challenge, with effective treatments being a top priority. Chemotherapy, while common, has significant limitations like severe side effects and poor drug efficiency. Gene therapy, though promising, also faces challenges such as instability and off-target effects. Poly(amino acid) carriers, due to their biocompatibility and degradability, show potential for improving drug delivery, reducing chemotherapy toxicity, and enhancing gene therapy effectiveness (Hu et al. 2023).

### Cytotoxicity analyses

A previous study emphasizes the importance of selectivity in the effectiveness of natural product-derived anticancer agents, suggesting that compounds with minimal effects on healthy cells are more desirable for therapeutic use (Newman and Cragg 2020). Furthermore, the higher cytotoxicity observed with CH_2_Cl_2_ and MeOH extracts against the HDFa cells and notably more so against the U87MG glioblastoma cell line might underscore the potential of these extracts as sources of anticancer agents. This differential cytotoxicity is crucial, as it suggests that CH_2_Cl_2_ and MeOH extracts might contain bioactive compounds with specific activity against cancer cells. The concept of using plant extracts with higher selectivity toward cancer cells, as evidenced by the marked cytotoxicity of CH_2_Cl_2_ and MeOH extracts on U87MG cells, is supported by a previous study where the potential of natural products was highlighted in targeting cancer cells with minimal impact on normal cells (Huang et al. 2021).

The finding that there was no significant increase in nuclear anomalies in the treated groups compared to the untreated control group suggests that the *H. scabrum* extracts, at the concentration tested, do not induce substantial DNA or chromosomal damage. This observation is particularly relevant in the context of previous research indicating the potential genotoxicity of various plant extracts that emphasizes the importance of evaluating plant extracts for genotoxic effects, as natural compounds can sometimes cause DNA damage leading to adverse health effects (Kirsch-Volders et al. 2003). Also, the absence of marked genotoxic effects in this study aligns with other research findings suggesting that some plant extracts may exert anticancer effects without causing significant damage to DNA. It was shown that certain herbal compounds could inhibit cancer cell growth through mechanisms that do not involve direct DNA damage, thereby offering a safer profile for therapeutic use (Choudhari et al. 2020).

This remarkable selectivity is indicative of the potential these extracts hold as targeted anticancer agents. The importance of selectivity lies in its potential to differentiate effectively between target and non-target cells or organisms. This characteristic is particularly valuable in therapeutic contexts, where the goal is to eliminate harmful cells, such as cancerous cells, without damaging healthy cells. High selectivity ensures treatments are more focused and effective, reducing the risk of adverse effects and improving patient outcomes by sparing healthy tissues from unintended harm (Aldrich et al. 2022).

The non-genotoxic nature of these extracts at the tested concentrations adds to their appeal as potential treatments. Future research should aim to identify the active compounds within these extracts, elucidate their mechanisms of action, and assess their efficacy in vivo (Gordaliza 2007).

### Enzyme inhibition assays

A study explored the pharmacological potential of three Turkish *Hypericum* species (*H. olympicum*, *H. pruinatum*, and *H. scabrum*) by assessing the inhibitory activity of their methanolic extracts on key physiological enzymes. The enzymes studied included cholinesterases (AChE and BChE), *α*-amylase, and *α*-glucosidase (involved in diabetes). For *α*-glucosidase inhibition, *H. scabrum* exhibited moderate activity with a value of 20.62 mmol ACAE per g extract, which was similar to that of *H. olympicum* (20.06 mmol ACAE per g extract) and *H. pruinatum* (20.45 mmol ACAE per g extract). In terms of *α*-amylase inhibition, *H. scabrum* showed a moderate effect with an inhibition value of 0.51 mmol ACAE per g extract**,** which was comparable to *H. pruinatum* (0.49 mmol ACAE per g extract), but slightly higher than *H. olympicum* (0.61 mmol ACAE per g extract). Regarding anticholinesterase activity, *H. scabrum* demonstrated modest inhibition of AChE with a value of 1.83 mg GALAE per g extract, slightly lower than *H. pruinatum* (2.03 mg GALAE per g extract) and comparable to *H. olympicum* (1.85 mg GALAE per g extract). For BChE, *H. scabrum* exhibited lower activity with an inhibition of 0.56 mg GALAE per g extract, which was less potent than both *H. pruinatum* (1.26 mg GALAE per g extract) and *H. olympicum* (0.35 mg GALAE per g extract) (Llorent-Martínez et al. 2018). In a study, it was aimed to explore the phytochemical composition of *H. scabrum* extracts and evaluate their significant biological activities, particularly focusing on enzyme inhibition. Regarding enzyme inhibition, the methanol extract showed the most promising results for *α*-glucosidase inhibition (IC_50_ 1.88 µg/mL) and AChE inhibition (IC_50_ 8.41 µg/mL), indicating potential antidiabetic and neuroprotective effects (Altay et al. 2022).

Our study represented the most comprehensive research conducted on *H. scabrum* to date. It is the first to extensively investigate the effects of extracts, prepared from various plant parts with differing polarities, on a wide range of enzymes.

The findings of this study provide not only pharmacological insights but also an opportunity to understand the ecological and evolutionary significance of the bioactive compounds in *Hypericum scabrum*. The accumulation of high levels of phenolic acids such as quinic acid, chlorogenic acid, and vanillic acid may serve as an adaptive strategy to mitigate environmental stressors. Phenolic compounds are well documented for their role in plant defense mechanisms, protecting against herbivory, pathogen attacks, and oxidative stress caused by UV radiation or drought. The antioxidant properties observed in the extracts likely reflect this ecological function, which has been co-opted for potential therapeutic applications. From an evolutionary perspective, the rich phytochemical diversity of *H. scabrum* might result from adaptive radiation or local adaptation to its ecological niche. These bioactive compounds not only provide a defense against biotic and abiotic challenges but may also play a role in ecological interactions, such as competition or facilitation with other species in the community. The morphological and anatomical characteristics observed, such as the presence of secretory trichomes and amphistomatic leaves, further highlight the ecological adaptations of the plant. Trichomes may serve dual roles in both the secretion of defensive compounds and the reduction of water loss, crucial for survival in arid environments where *H. scabrum* is typically found.

In conclusion, the bioactive properties of *H. scabrum* not only underscore its therapeutic potential but also reveal its evolutionary strategies for survival and reproduction. Future research should explore the ecological roles of these compounds in situ and investigate how environmental factors influence their biosynthesis. Understanding these connections could provide deeper insights into the interplay between ecology, evolution, and phytochemistry.

## Conclusion

In conclusion, this study offers valuable insights into the biochemical properties and therapeutic potential of *H. scabrum.* Comprehensive biochemical analysis revealed a rich profile of bioactive compounds. ICP-MS analysis identified significant concentrations of sodium and potassium, while LC–MS/MS profiling uncovered key metabolites such as quinic acid and proline, with proline reaching an impressive concentration of 494.0482 nmol/mL in the aerial part extract. These findings underscore the plant’s potential as a source of bioactive substances with diverse therapeutic effects. The methanol extract displayed potent inhibitory activity against key enzymes involved in diabetes and neurodegenerative diseases, including *α*-glucosidase, with an IC_50_ value of < 1 µg/mL, surpassing the efficacy of the standard drug acarbose. Furthermore, the anticancer assays demonstrated selective cytotoxicity, with higher activity against glioblastoma cells compared to fibroblasts, suggesting *H. scabrum*’s potential in cancer treatment. These results emphasize *H. scabrum*’s promising therapeutic prospects, particularly in the management of diabetes, neurodegenerative diseases, and cancer. Further investigations into its biochemical composition and mechanisms of action will pave the way for developing innovative, plant-based therapeutic strategies.

## Data Availability

Samples of the extracts are available from the authors.
